# Supplementary material for: Low-dose radiotherapy combined with dual PD-L1 and VEGFA blockade elicits antitumor response in hepatocellular carcinoma mediated by activated intratumoral CD8+ exhausted-like T cells
Source: Nat Commun. 2023 Nov 24;14:7709. doi: 10.1038/s41467-023-43462-1 (PMC10673920; doi:10.1038/s41467-023-43462-1)
Supplement: Supplementary file 1 — Supplementary Information [file 41467_2023_43462_MOESM1_ESM.pdf]

**Title:**

Low-dose Radiotherapy Combined with Dual PD-L1 and VEGFA Blockade Elicits Antitumor Response in Hepatocellular Carcinoma Mediated by Activated Intratumoral CD8$^{+}$ exhausted-like T cells

**Supplementary Figures:**

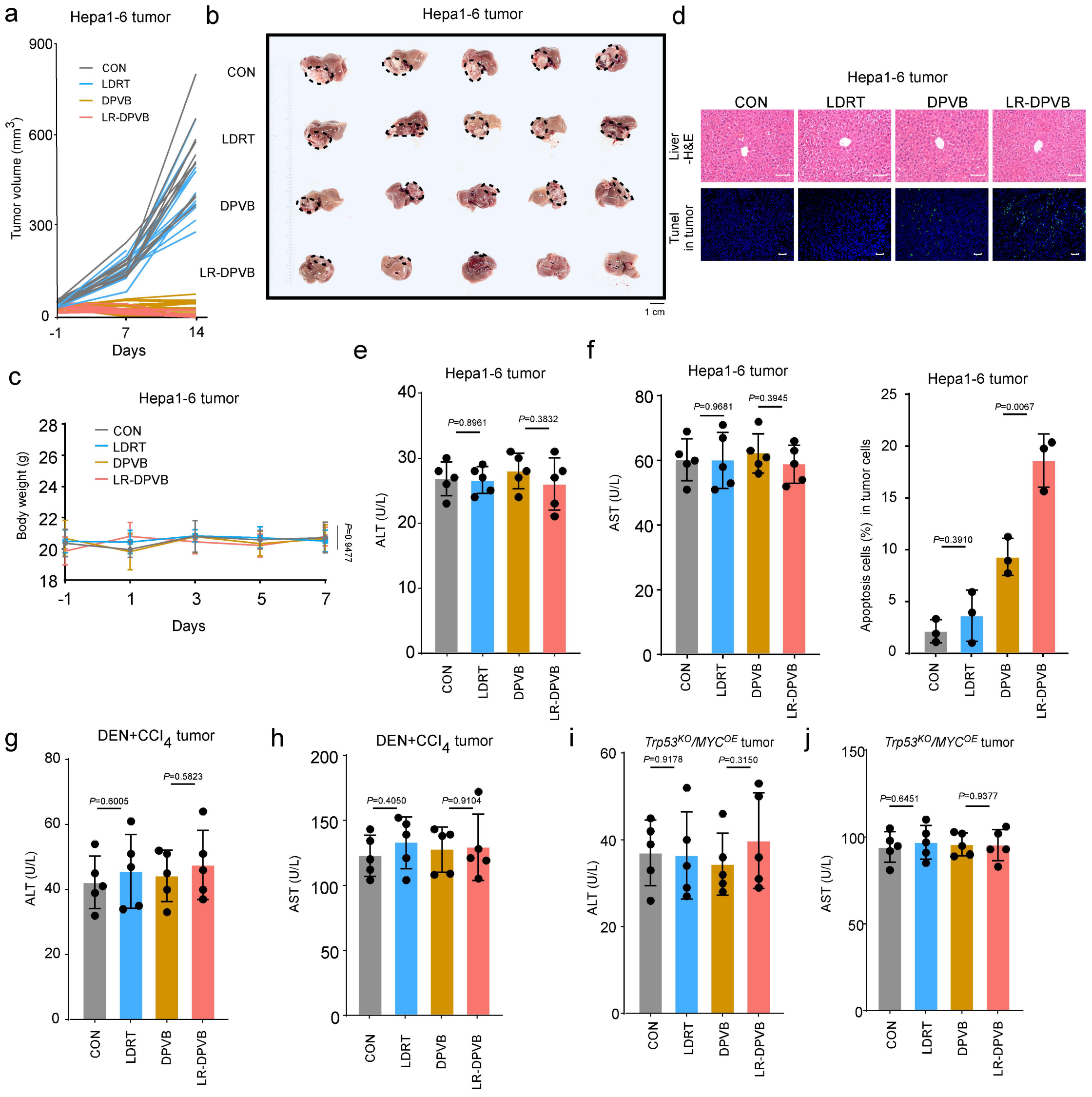


**Supplementary Fig. 1 Safety evaluations of combined local liver LDRT in combined with DPVB.**

**(a)** Tumor growth curves of Hepa1-6 HCC model during the therapeutic cycle evaluated by MRI. n=14 mice/group. **(b)** Representative image of Hepa1-6 HCC model at the end of the second therapeutic cycles (circled by black lines). n=5 mice/ group. **(c)** Body weight changes of Hepa1-6 HCC model mice during the two therapeutic cycles. n=5 mice/group. *P* values were calculated using One-way

repeated-measures ANOVA test. **(d)** H&E staining for liver from Hepa1-6 HCC model mice (up) and Tunel assay of Hepa1-6 HCC model liver tumor (down) in each group at the end of the second therapeutic cycles. Quantification of Tunel positive cells in each group. Scale bars: 100 μm. **(e-f)** Serum levels of alanine transaminase (ALT), aspartate aminotransferase (AST) in Hepa1-6 HCC model mice were measured at the end of the second therapeutic cycles. **(g-h)** Serum levels of alanine transaminase (ALT), aspartate aminotransferase (AST) in DEN+$CCl_4$ HCC model mice were measured at the end of the therapeutic cycles. **(i-j)** Serum levels of alanine transaminase (ALT), aspartate aminotransferase (AST) in $Trp53^{KO}/MYC^{OE}$ HCC model mice were measured at the end of the therapeutic cycles. Data of **(d)** shown as means ± SD derived from tumor mouse models (n=3 mice/group). Data of **(e-f)** and **(g-j)** shown as means ± SD derived from tumor mouse models (n=5 mice/group). *P* values of **(d-j)** were calculated using a two-sided unpaired Student's *t* test. Source data are provided as a Source Data file.

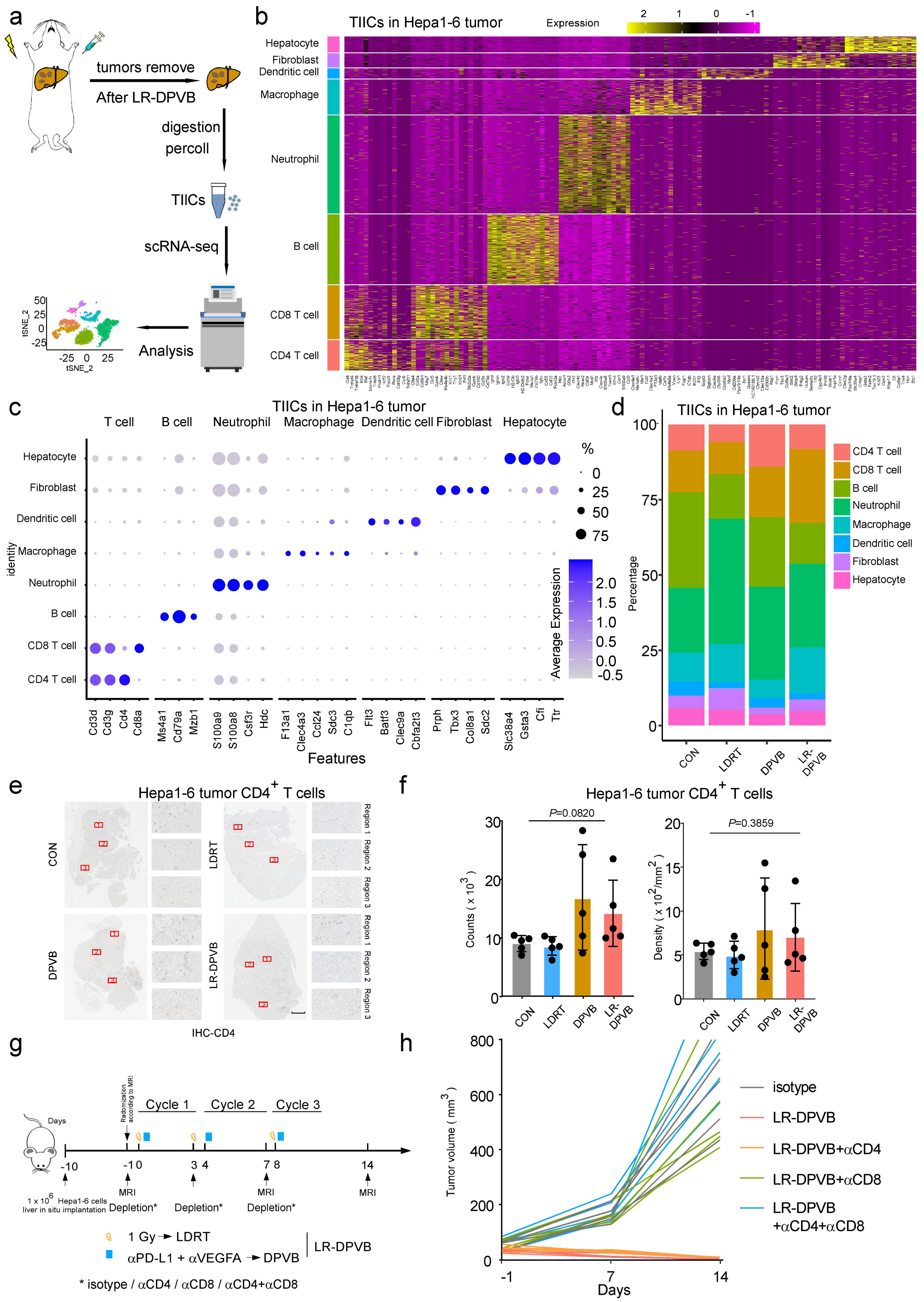


**Supplementary Fig. 2 The scRNA-seq characterization of TIICs isolated from Hepa1-6 HCC tumors with 4 different treatment.**

**(a)** The flow chart of single cells isolation and scRNA-seq. n=3 mice/group. **(b)**

Heatmap of display of cell identity marker genes. n=3 mice/group. **(c)** TIICs expressing indicated genes across major cell populations and their corresponding average expression (size of dot indicates the percentage of cells in each population; expression intensity is indicated by color). n=3 mice/group. **(d)** Percentage stack plot representing the proportion of each cell population in different groups. n=3 mice/group. **(e-f)** Representative images **(e)** and quantification **(f)** of CD4 IHC staining in serial sections. n=5 mice/group. *P* values were calculated using a two-sided unpaired Student's *t* test. Data shown as means ± SD. Scale bars: 500 μm. **(g)** Schematic depiction of $CD4^+$ and/or $CD8^+$ T cell depletion experiment in Hepa1-6 bearing LR-DPVB treated mice. **(h)** Tumor growth was assessed at the indicated days. n=5 mice/group. Source data are provided as a Source Data file.

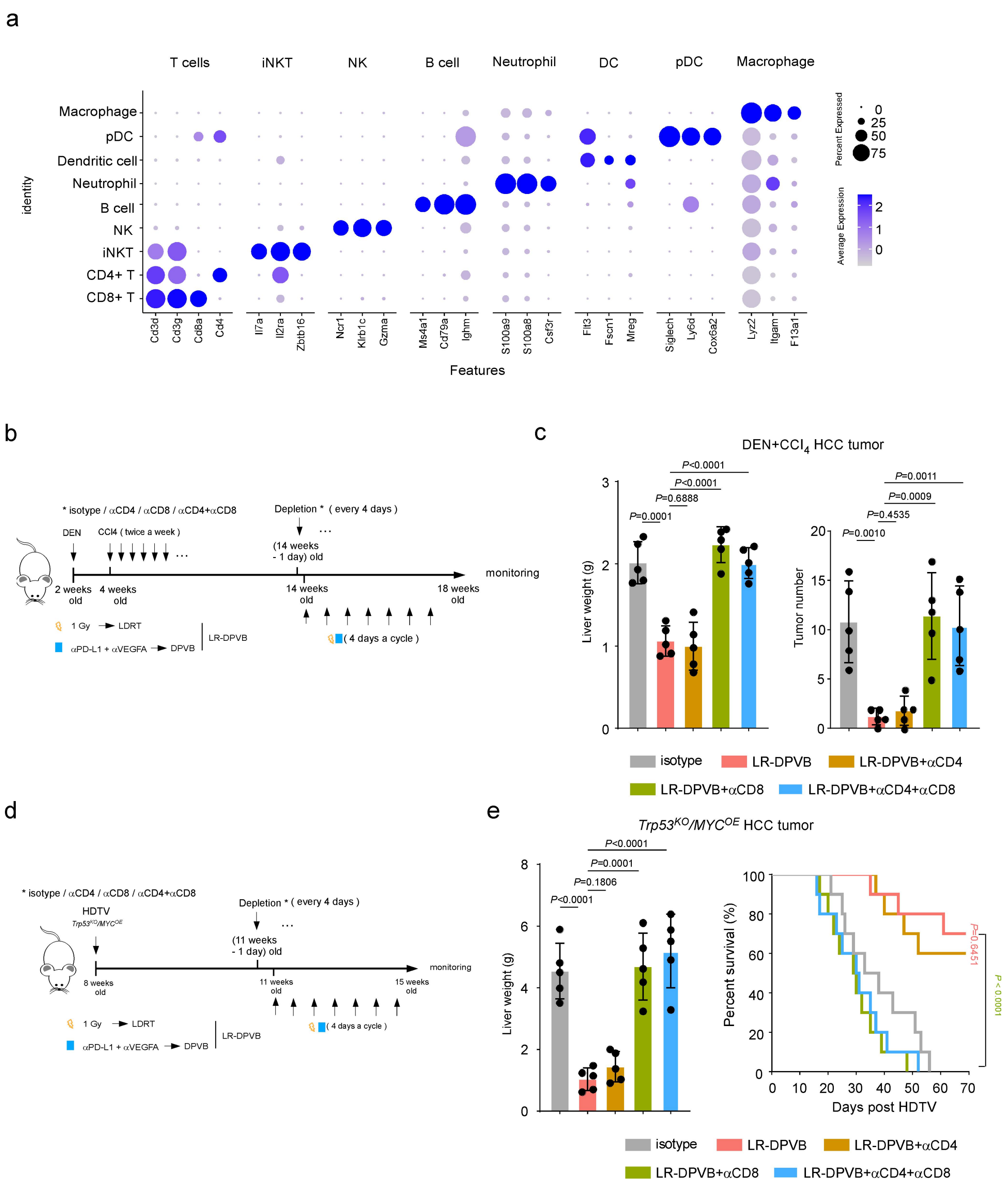


**Supplementary Fig. 3 The scRNA-seq characterization of CD45$^+$ TIICs isolated from DEN+CCl$_4$ HCC tumors with 4 different treatment.**

**(a)** CD45$^+$ TIICs expressing indicated genes across major cell populations and their corresponding average expression (size of dot indicates the percentage of cells in each population; expression intensity is indicated by color). n=3 mice/group. **(b)** Schematic depiction of CD4$^+$ and/or CD8$^+$ T cell depletion experiment in DEN+CCl$_4$ HCC mice. **(c)** The liver weight (left panel) and tumor number (right panel) of DEN+CCl$_4$ HCC model at the indicated groups. n=5 mice/group. *P* values were calculated using a

two-sided unpaired Student's *t* test. **(d)** Schematic depiction of $CD4^+$ and/or $CD8^+$ T cell depletion experiment in *Trp53*$^{KO}$/*MYC*$^{OE}$ HCC mice. **(e)** The liver weight (left panel, n=5 mice/group, *P* values were calculated using a two-sided unpaired Student's *t* test) and Kaplan – Meier curve (right panel, n=10 mice/group, *P* values were determined by log-rank test (Mantel-Cox)) of *Trp53*$^{KO}$/*MYC*$^{OE}$ HCC model at the indicated groups. Data of **(c)** and **(e)** shown as means ± SD of derived from tumor mouse models (n=5 mice/group). Source data are provided as a Source Data file.

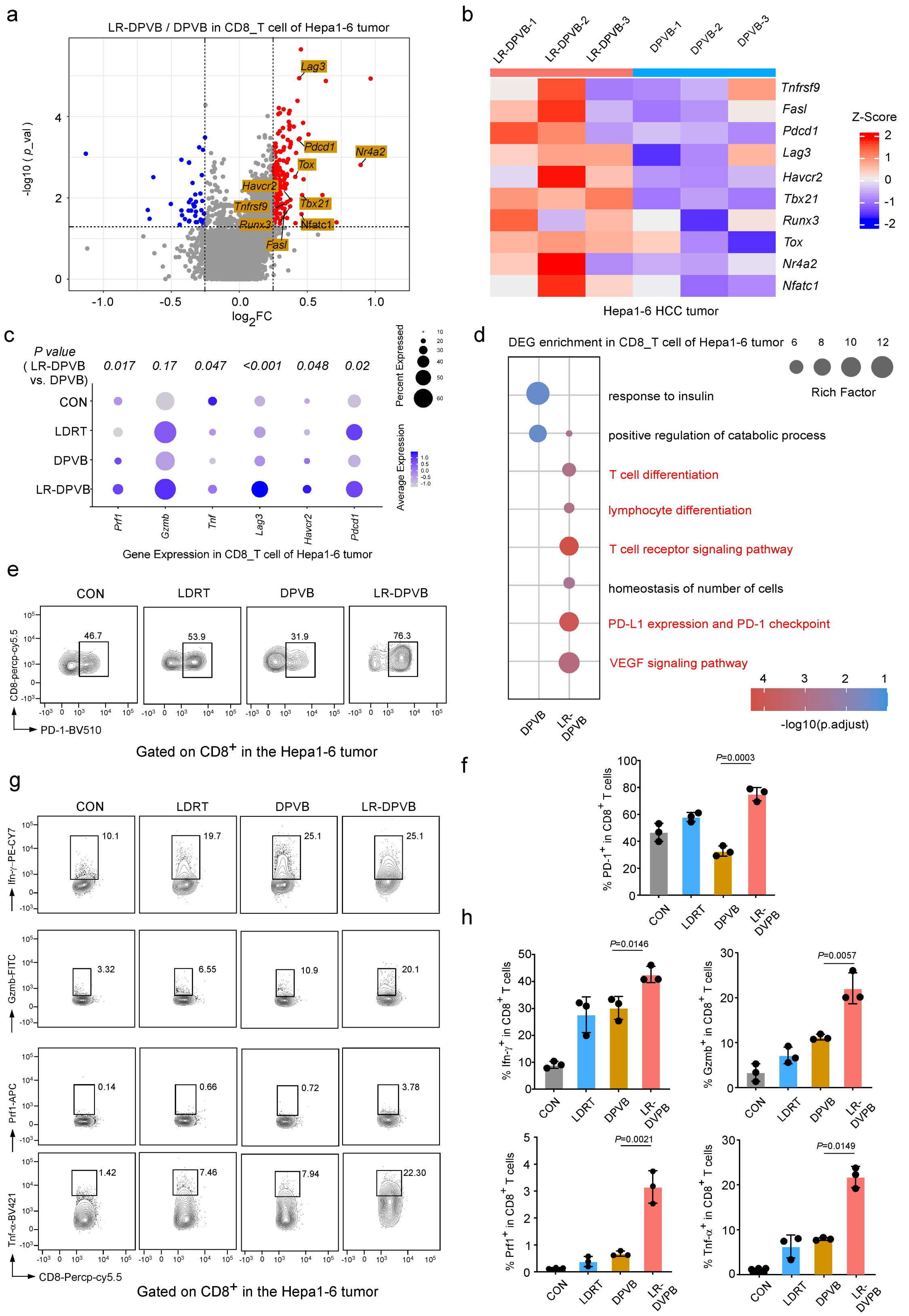


**Supplementary Fig. 4 The LR-DPVB expands the exhausted and activated of $CD8^+$ T cells in Hepa1-6 HCC tumors.**

**(a)** Volcano plot representing the differentially expressed genes (DEGs) of CD8_T

cells in Hepa1-6 HCC tumor of LR-DPVB *vs* DPVB, the exhausted and activated associated molecular have been highlighted. The threshold of the DEGs is $|\log_2 FC| > 0.25$, $P < 0.05$. Red = upregulated genes; blue = downregulated genes; gray = non-DEGs. **(b)** Heatmaps of the exhausted and activated associated molecular depicting gene expression patterns across the LR-DPVB and DPVB groups in Hepa1-6 HCC tumor. **(c)** Dot gram representing the expression of various exhausted and effected function markers in CD8_T subsets in Hepa1-6 HCC tumor of indicated group. **(d)** Bubble chart of DEGs pathway enrichment of CD8_T cells in Hepa1-6 HCC tumor with LR-DPVB and DPVB groups. **(e-f)** Representative flow cytometry plots **(e)** and quantification **(f)** of % PD-$1^+$ in $CD8^+$ T cells of Hepa1-6 HCC tumor in indicated groups. **(g-h)** Representative flow cytometry plots **(g)** and quantification **(h)** of % $Prf1^+$, % $Gzmb^+$, % Tnf-$\alpha^+$ and % Ifn-$\gamma^+$ in $CD8^+$ T cells of Hepa1-6 HCC tumor in indicated groups. Data of **(f)** and **(h)** shown as means ± SD derived from tumor mouse models (n=3 mice/group). *P* values of **(f)** and **(h)** were calculated using a two-sided unpaired Student's *t* test. **(a-d)** n=3 mice/group. Source data are provided as a Source Data file.

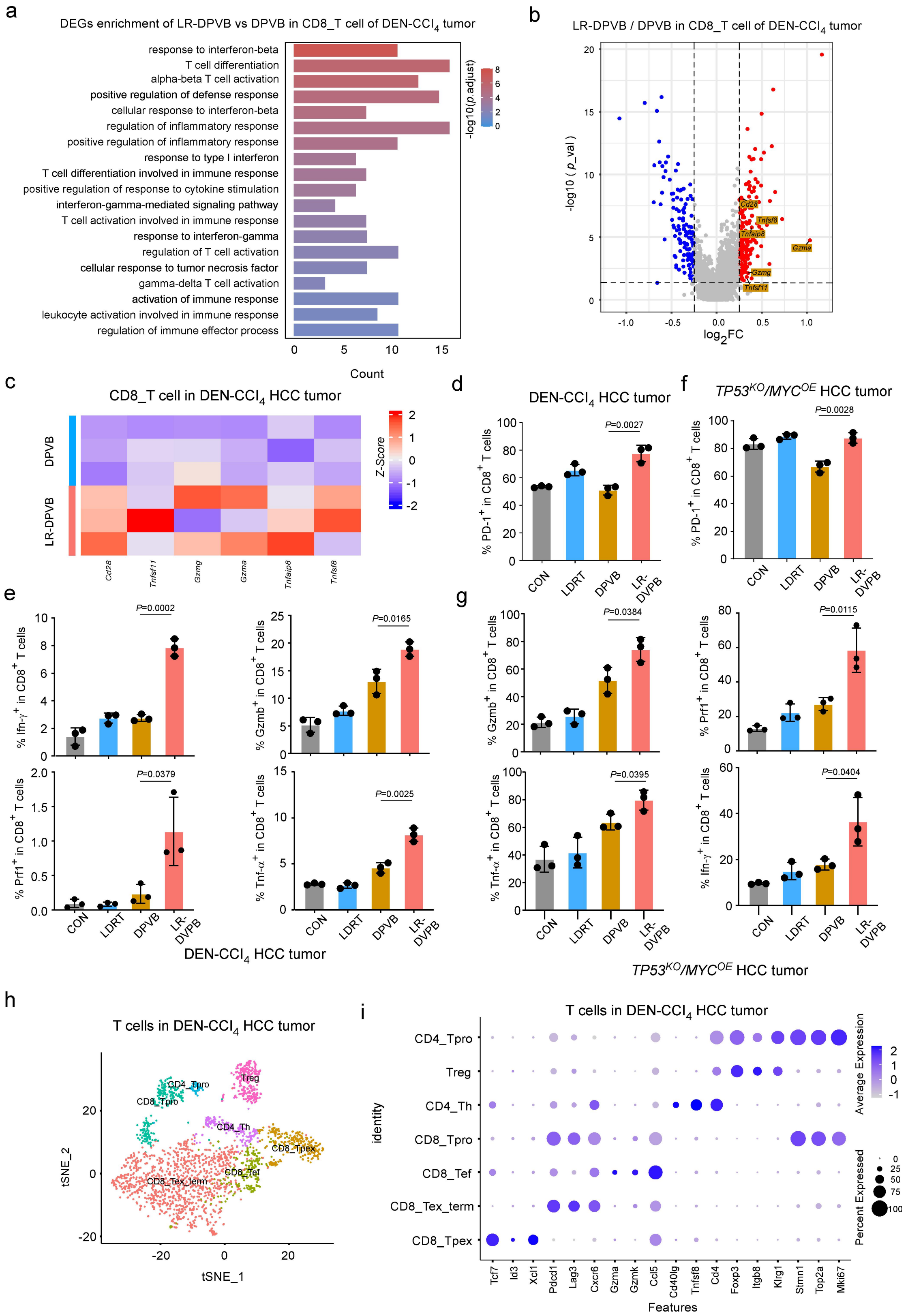

**Supplementary Fig. 5 LR-DPVB enlarged the effector function and cytolytic capacity of tumor-rejecting CD8_T cell in DEN+CCI₄ HCC tumors and *Trp53^{KO}/MYC^{OE}* HCC tumors.**

**(a)** Bar chart of DEGs pathway enrichment of CD8_T cell in DEN+CCI₄ HCC tumor

with LR-DPVB and DPVB groups. **(b-c)** Vocano map **(b)** and heatmap **(c)** of the activated associated molecular depicting gene expression patterns in CD8_T cell across the LR-DPVB and DPVB groups in DEN+$CCI_4$ HCC tumor. **(d)** Quantification of % PD-$1^+$ in $CD8^+$ T cells of DEN+$CCI_4$ HCC tumor in indicated groups. **(e)** Quantification of% $Prf1^+$ , % $Gzmb^+$ , % Tnf-$\alpha^+$ and % Ifn-$\gamma^+$ in $CD8^+$ T cells of DEN+$CCI_4$ HCC tumor in indicated groups. **(f)** Quantification of % PD-$1^+$ in $CD8^+$ T cells of $Trp53^{KO}/MYC^{OE}$ HCC tumor in indicated groups. **(g)** Quantification of% $Prf1^+$ , % $Gzmb^+$ , % Tnf-$\alpha^+$ and % Ifn-$\gamma^+$ in $CD8^+$ T cells of $Trp53^{KO}/MYC^{OE}$ HCC tumor in indicated groups. **(h)** tSNE maps of scRNAseq data of T cell from DEN+$CCI_4$ HCC tumor (n=3 tumors per group). CD4_Tpro = proliferating CD4_T cell, Treg = CD4_Treg, CD4_Th = helper CD4_T cell, CD8_Tpro = proliferating CD8_T cell, CD8_Tef = transient effected CD8_T cell, CD8_Tex_term = terminally exhausted CD8_T cell, CD8_Tpex = progenitor exhausted CD8_T cell. **(i)** DEN+$CCI_4$ HCC tumor T cells expressing indicated genes across major cell subsets and their corresponding average expression (size of dot indicates the percentage of cells in each population; expression intensity is indicated by color). **(a-c)** n=3 mice/group. Data of **(d-g)** shown as means ± SD derived from tumor mouse models (n=3 mice/group). *P* values of **(d-g)** were calculated using a two-sided unpaired Student's *t* test. Source data are provided as a Source Data file.

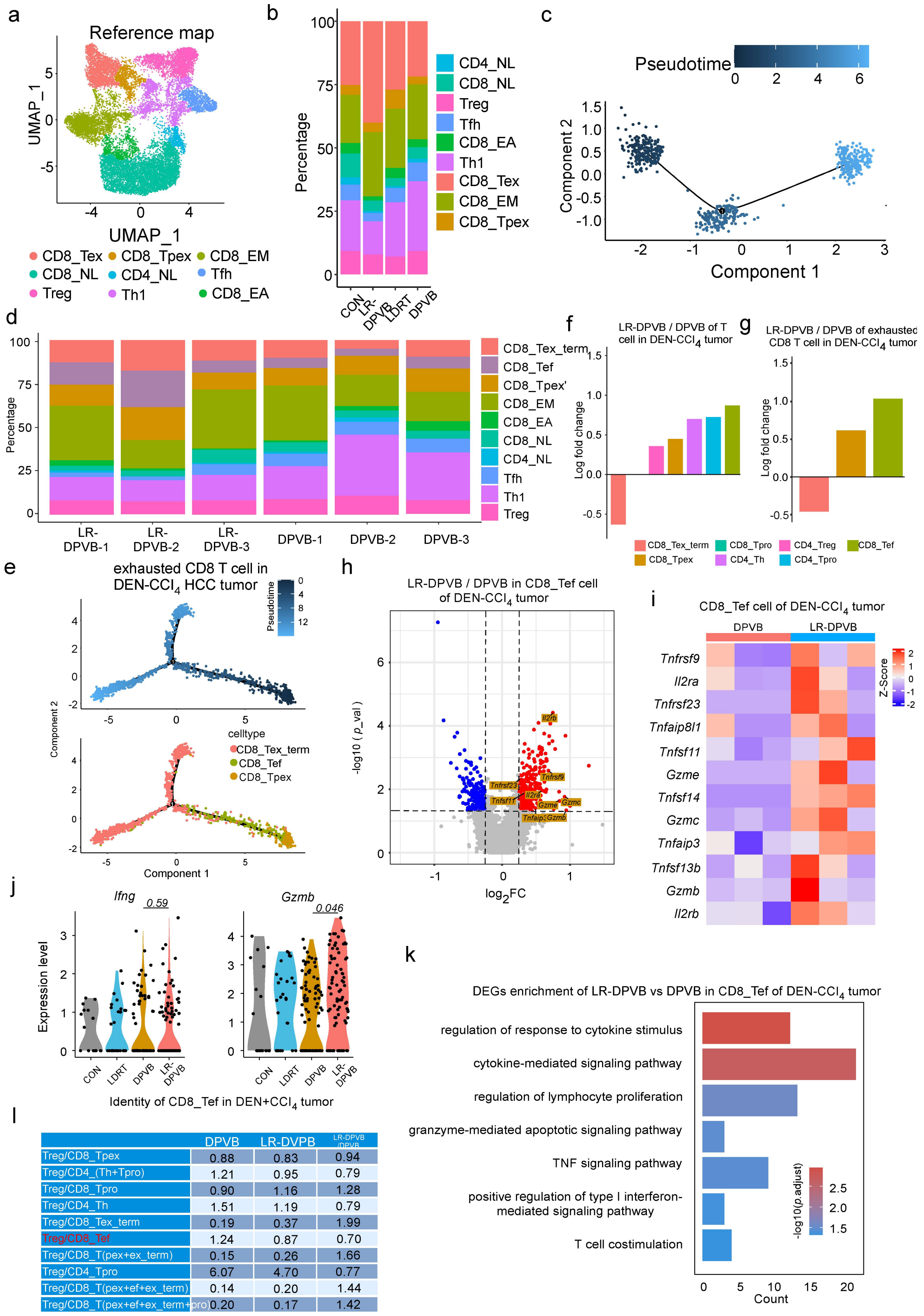


| | DPVB | LR-DVPB | LR-DPVB /DPVB |
|---|---|---|---|
| Treg/CD8_Tpex | 0.88 | 0.83 | 0.94 |
| Treg/CD4_(Th+Tpro) | 1.21 | 0.95 | 0.79 |
| Treg/CD8_Tpro | 0.90 | 1.16 | 1.28 |
| Treg/CD4_Th | 1.51 | 1.19 | 0.79 |
| Treg/CD8_Tex_term | 0.19 | 0.37 | 1.99 |
| Treg/CD8_Tef | 1.24 | 0.87 | 0.70 |
| Treg/CD8_T(pex+ex_term) | 0.15 | 0.26 | 1.66 |
| Treg/CD4_Tpro | 6.07 | 4.70 | 0.77 |
| Treg/CD8_T(pex+ef+ex_term) | 0.14 | 0.20 | 1.44 |
| Treg/CD8_T(pex+ef+ex_term+pro) | 0.20 | 0.17 | 1.42 |

**Supplementary Fig. 6 The LR-DPVB expands the transitory effected CD8$^{+}$ T cells (CD8_Tef) featured a dramatic enrichment of effector function and cytolytic capacity.**

**(a)** Reference map of UMAP plots of T cells scRNA-seq data (n=3 Hepa1-6 HCC

tumors per treatment). **(b)** Percentage stack plot representing the proportion of each cell subsets in different groups of Hepa1-6 HCC tumor. **(c)** Pseudotime trajectory analysis of CD8_Tpex and CD8_Tex clusters in Hepa1-6 was performed by the Monocle tool. **(d)** Percentage stack plot representing the proportion of each cell subsets in individual Hepa1-6 HCC tumor (no.1-3) from LR-DPVB or DPVB. (e) Pseudotime trajectory analysis of CD8_Tpex, CD8_Tef and CD8_Tex_term clusters of DEN+$CCl_4$ HCC tumor was performed by the Monocle tool. **(f)** Fold-change of T cell subsets of DEN+$CCl_4$ HCC tumor following LR-DPVB vs DPVB. **(g)** Fold-change of CD8_Tex subsets of DEN+$CCl_4$ HCC tumor following LR-DPVB vs DPVB. **(h-i)** Vocano map **(h)** and heatmap **(i)** of the activated associated molecular depicting gene expression patterns in CD8_Tef across the LR-DPVB and DPVB groups in DEN+$CCl_4$ HCC tumor. **(j)** Violin plot representing the expression of various exhausted and effected function markers in CD8_Tef subsets in DEN+$CCl_4$ HCC tumor of indicated group. **(k)** Bar chart of DEGs pathway enrichment of CD8_Tef cell in DEN+$CCl_4$ HCC tumor with LR-DPVB and DPVB groups. **(l)** Table describes Treg: CD8 or CD4 ratios in DEN+$CCl_4$ HCC tumor of LR-DPVB and DPVB groups.

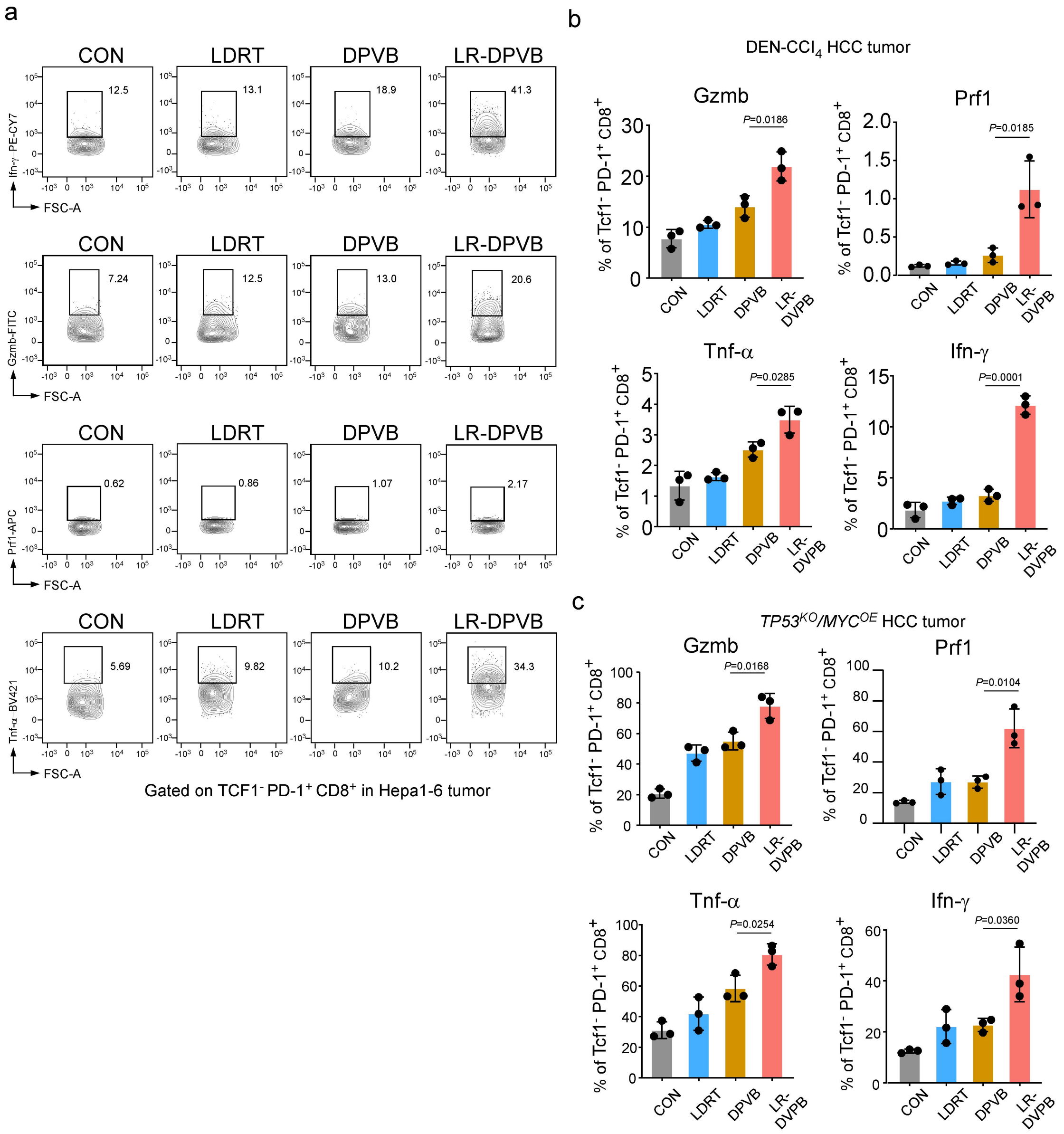


**Supplementary Fig. 7 The flow cytometry assays of intratumoral CD8_Tef in three HCC models.**

**(a)** Representative flow cytometry plots of % Prf1$^+$, % Gzmb$^+$, % Tnf-α$^+$ and % Ifn-γ$^+$ in TCF1$^-$ PD-1$^+$ CD8$^+$ T cells of Hepa1-6 tumor in the indicated groups. **(b)** Quantification of flow cytometry plots of % Prf1$^+$, % Gzmb$^+$, % Tnf-α$^+$ and % Ifn-γ$^+$ in TCF1$^-$ PD-1$^+$ CD8$^+$ T cells of DEN+$CCl_4$ HCC tumor in the indicated groups. **(c)** Quantification of flow cytometry plots of % Prf1$^+$, % Gzmb$^+$, % Tnf-α$^+$ and % Ifn-γ$^+$ in TCF1$^-$ PD-1$^+$ CD8$^+$ T cells of *Trp53$^{KO}$/MYC$^{OE}$* HCC tumors in the indicated groups. Data of **(b-c)** shown as means ± SD derived from tumor mouse models (n=3 mice/group). *P* values of **(b-c)** were calculated using a two-sided unpaired Student's *t*

test. Source data are provided as a Source Data file.

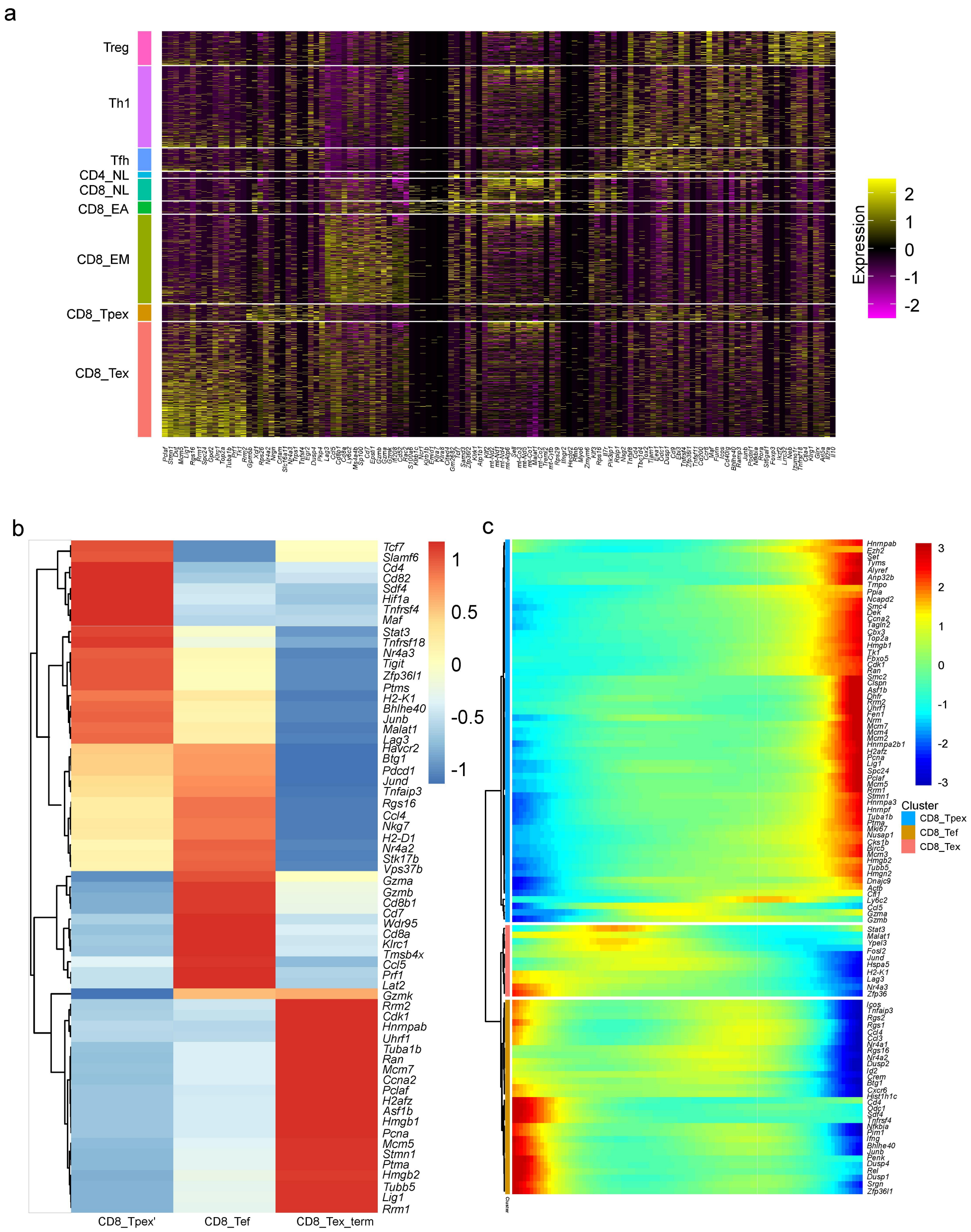


**Supplementary Fig. 8 Phenotypic characterization of intratumoral T cells.**

**(a)** The FindAllMarker function was used to calculate the tag genes of the classified cells, and the heatmap showed the top 15 tag genes of each subset. **(b)** The heatmap shows the characteristic genes of three cell subsets on the pseudotime trajectory. **(c)** The heatmap presents the top 100 genes associated with the pseudotime trajectory. **(a-c)** n=3 mice/group.

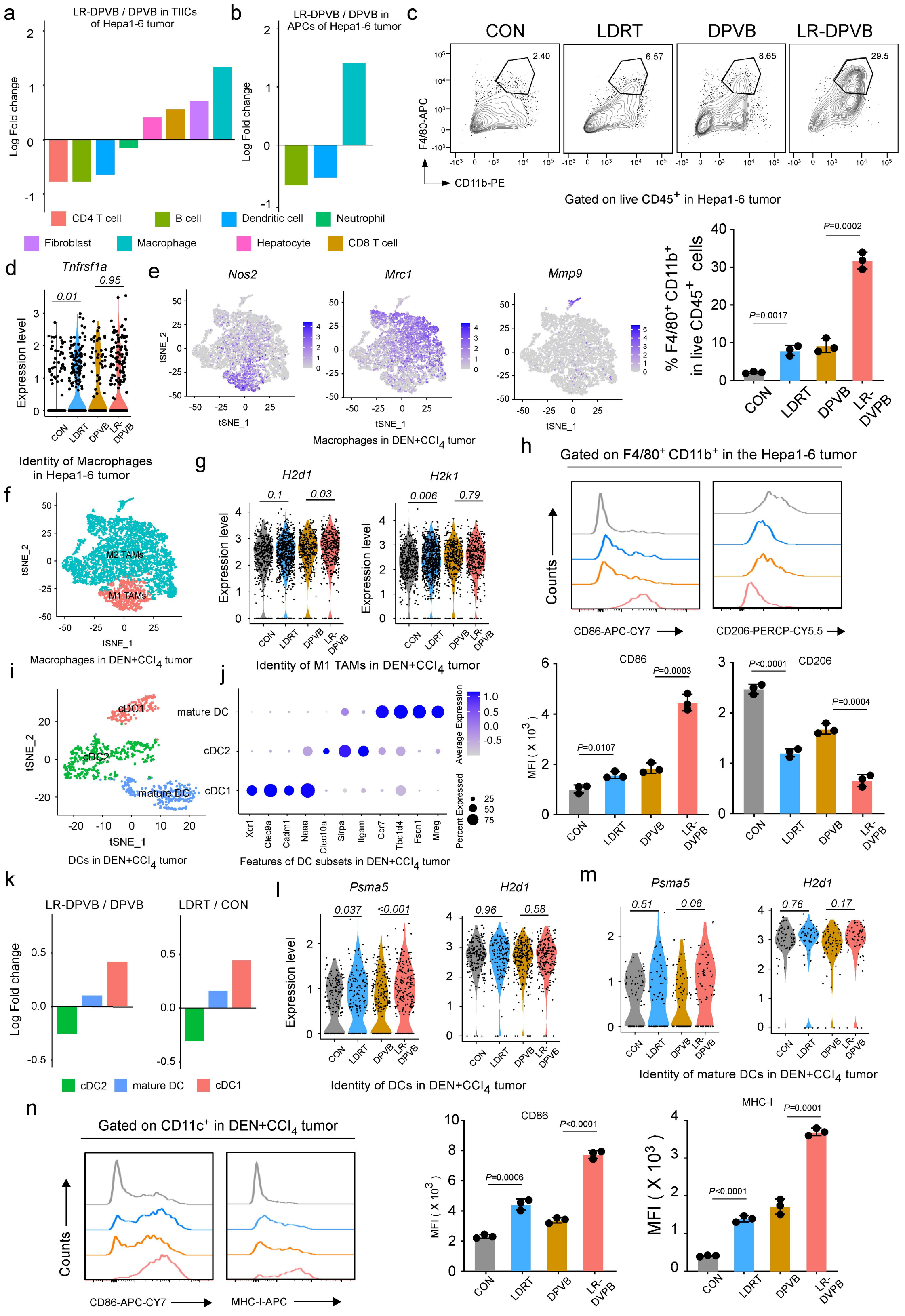

**Supplementary Fig. 9 LR-DPVB effectively enhanced the activation of the tumor's innate immune microenvironment.**

**(a)** Fold-change of TIICs populations from LR-DPVB and DPVB groups in Hepa1-6 HCC tumor. **(b)** Fold-change of antigen presented cell populations (APCs) from

LR-DPVB and DPVB groups in Hepa1-6 HCC tumor. **(c)** Representative (up panel) and quantification (down panel) of flow cytometry plots of % F4/80$^+$ CD11b$^+$ in live CD45$^+$ cells of Hepa1-6 HCC tumor TIICs at the end of the therapeutic cycle. **(d)** Violin plots representing the expression of *Tnfrsf1a* in macrophage subsets of Hepa1-6 HCC tumor with indicated treatment. **(e)** tSNE map of the indicated marker in macrophages of DEN+$CCI_4$ tumor. **(f)** tSNE map of the M1-type and M2-type macrophages of DEN+$CCI_4$ tumor. **(g)** Violin plots representing the expression of *H2d1* and *H2k1* in M1-type tumor-associated macrophage (TAMs) of DEN+$CCI_4$ tumor with indicated treatment. **(h)** Expression of CD86 and CD206 of F4/80$^+$ CD11b$^+$ cells in the Hepa1-6 HCC tumor with indicated treatment. Data shown as means ± SD of three independent experiments. **(i)** tSNE maps of scRNAseq data from DCs of DEN+$CCI_4$ tumor (n=3 per group). **(j)** DCs expressing indicated genes across major cell populations and their corresponding average expression (size of dot indicates the percentage of cells in each population; expression intensity is indicated by color). **(k)** Fold-change of DCs populations from LR-DPVB and DPVB groups (left panel) and LDRT and CON groups (right panel) in DEN+$CCI_4$ tumor. **(l-m)** Violin plots representing the expression of *Psma5* and *H2d1* in DCs **(l)** and mature DCs **(m)** of DEN+$CCI_4$ tumor with indicated treatment. **(n)** Expression of CD86 and MHC-I of CD11c$^+$ cells in the Hepa1-6 HCC tumor with indicated treatment. **(a-n)** n=3 mice/group. Data of **(c)**, **(h)** and **(n)** shown as means ± SD derived from tumor mouse models (n=3 mice/group). *P* values of **(c)**, **(h)** and **(n)** were calculated using a two-sided unpaired Student's *t* test. Source data are provided as a Source Data file.

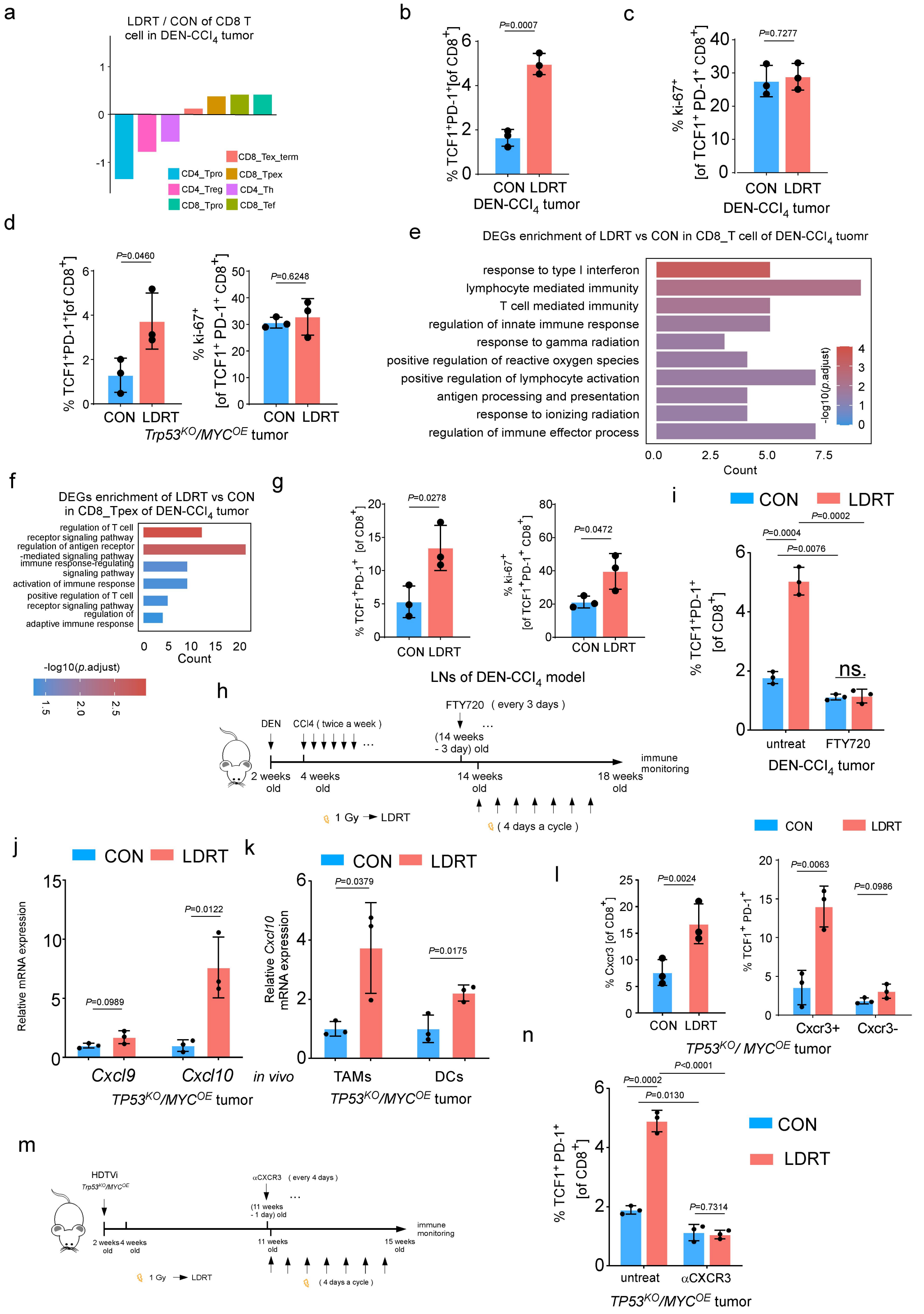


**Supplementary Fig. 10 LDRT-induced intratumoral stem-like CD8_Tpex was recruited from the dLNs through by CXCL10/CXCR3 axis.**

**(a)** Fold-change of T cell subsets following LDRT vs CON in DEN+CCI4 model. n=3 mice/group. **(b-c)** Flow cytometry quantification of % TCF1$^{+}$ PD-1$^{+}$ in CD8$^{+}$ T cells

and % Ki-67$^+$ in TCF1$^+$ PD-1$^+$ CD8$^+$ T cells of the indicated groups in DEN-CCl$_4$ model. **(d)** Flow cytometry quantification of % TCF1$^+$ PD-1$^+$ in CD8$^+$ T cells (left panel) and % Ki-67$^+$ in TCF1$^+$ PD-1$^+$ CD8$^+$ T cells (right panel) of the indicated groups in *Trp53$^{KO}$/MYC$^{OE}$* model. **(e)** Bar chart of DEGs pathway enrichment of CD8_T cell in DEN+CCl$_4$ HCC tumor with LDRT and CON groups. n=3 mice/group. **(f)** Bar chart of DEGs pathway enrichment of CD8_Tpex in DEN+CCl$_4$ HCC tumor with LR-DPVB and DPVB groups. n=3 mice/group. **(g)** Flow cytometry quantification of % TCF1$^+$ PD-1$^+$ in CD8$^+$ T cells and % Ki-67$^+$ in TCF1$^+$ PD-1$^+$ CD8$^+$ T cells of the dLNs of indicated groups in DEN-CCl$_4$ model. **(h-i)** Schematic FTY720 in DEN-CCl$_4$ LDRT treated mice **(h)** and quantification of % TCF1$^+$ PD-1$^+$ in CD8$^+$ T cells in the tumors of the indicated groups **(i)**. **(j)** Relative mRNA expression of *Cxcl9* and *Cxcl10* in LDRT and CON groups of *Trp53$^{KO}$/MYC$^{OE}$* tumor tissue. **(k)** Relative mRNA expression of *Cxcl10* in LDRT and CON groups of tumor-associated macrophages (TAMs, F4/80+ CD11b$^+$ CD45$^+$ L/D$^-$) and DCs (CD11c$^+$ CD45$^+$ L/D$^-$) cells sorted from tumor-infiltrated immune cells (TIICs) of *Trp53$^{KO}$/MYC$^{OE}$* tumor. **(l)** Quantification flow cytometry of % CXCR3$^+$ in CD8$^+$ T cells (left panel), % TCF1$^+$ PD-1$^+$ in CXCR3$^+$ CD8$^+$ T cells and CXCR3$^-$ CD8$^+$ T cells (right panel) in the *Trp53$^{KO}$/MYC$^{OE}$* tumors of the indicated groups. **(m-n)** Schematic depiction of anti-CXCR3 in LDRT-treated *Trp53$^{KO}$/MYC$^{OE}$* mice **(m)** and quantification of % TCF1$^+$ PD-1$^+$ in CD8$^+$ T cells in the tumors of the indicated groups **(n)**. Data of **(b-d)**, **(g)**, **(i-l)** and **(n)** shown as means ± SD derived from tumor mouse models (n=3 mice/group). *P* values of **(b-d)**, **(g)**, **(i-l)** and **(n)** were calculated using a two-sided unpaired Student's *t* test. Source data are provided as a Source Data file.

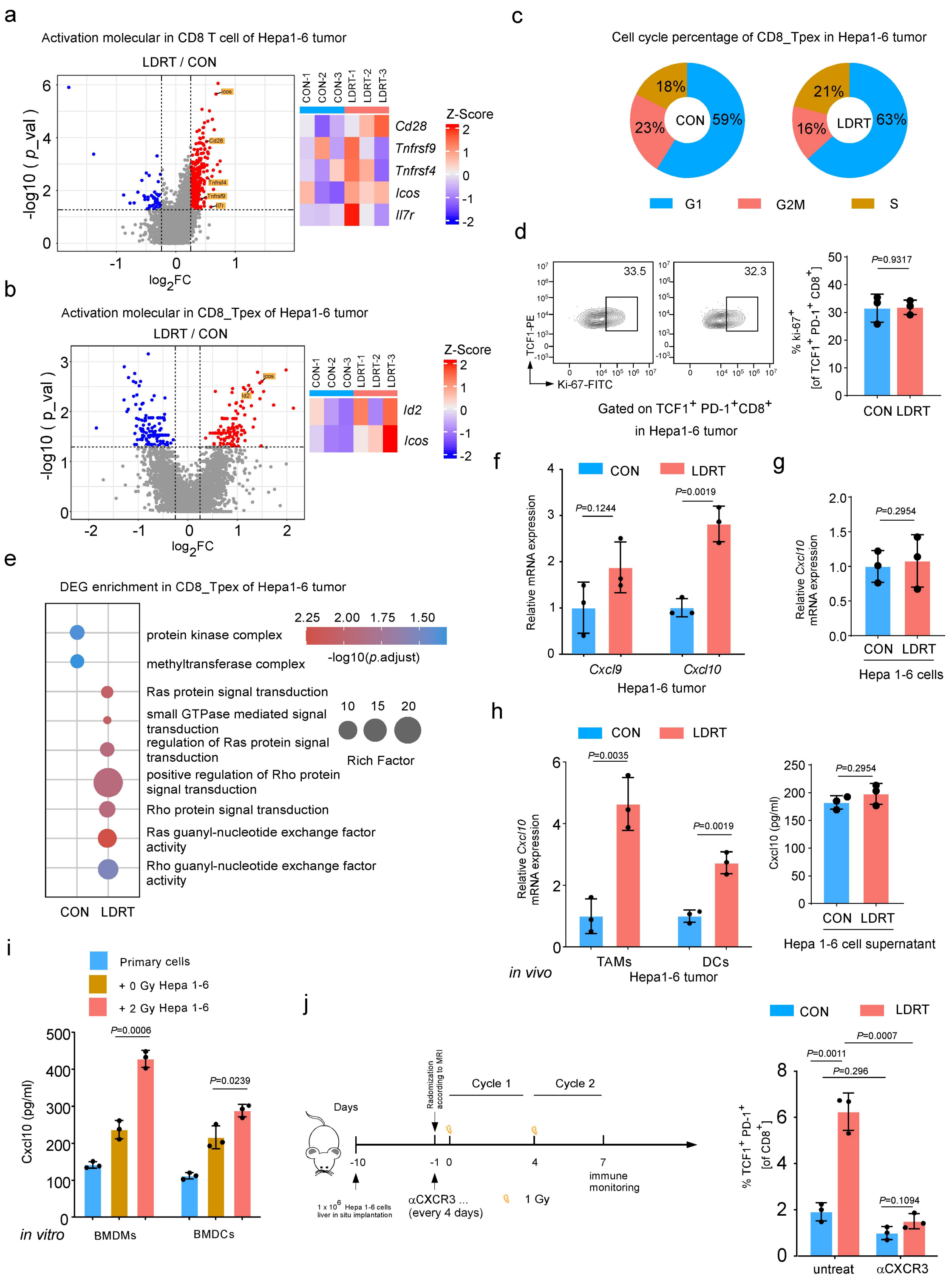

**Supplementary Fig. 11 Role of peripheral lymphatic trafficking in intratumoral stem-like CD8+ Tpex accumulation via CXCL10/CXCR3 axis.**

**(a-b)** Volcano plot representing the DEGs of LDRT vs CON in CD8 T cell **(a)** and CD8_Tpex **(b)**, the activation molecular have been highlighted (left panel). The threshold of the DEGs is $|log_2FC|>0.25$, $P<0.05$. Red = upregulated genes; blue = downregulated genes; gray = non-DEGs. Heatmaps (right panel) of the activation

molecular depicting gene expression patterns across the LDRT and CON groups in $CD8^+$ T cells **(a)** and CD8_Tpex **(b)**. n=3 mice/group. **(c)** Pie chart depicting the % of the cell cycle of CD8_Tpex in LDRT and CON groups. n=3 mice/group. **(d)** Representative flow cytometry plots (left panel) and quantification (right panel) of % Ki-$67^+$ in $TCF1^+$ PD-$1^+$ $CD8^+$ T cells of the indicated groups. **(e)** Bubble chart of DEG pathway enrichment of CD8_Tpex in LDRT and CON groups. n=3 mice/group. **(f)** Relative mRNA expression of *Cxcl9* and *Cxcl10* in LDRT and CON groups of Hepa1-6 tumor tissue. **(g)** Relative mRNA expression of *Cxcl10* in Hepa1-6 cells (up panel) and protein level of of Cxcl10 of supernatant of Hepa1-6 cells in LDRT and CON groups (down panel). **(h)** Relative mRNA expression of *Cxcl10* in LDRT and CON groups of tumor-associated macrophages (TAMs, F4/80+ $CD11b^+$ $CD45^+$ L/$D^-$) and DCs ($CD11c^+$ $CD45^+$ L/$D^-$) cells sorted from tumor-infiltrated immune cells (TIICs). **(i)** The protein level of of Cxcl10 of supernatant of the indicated groups of *in vitro* co-culture assays. **(j)** Schematic depiction of anti-CXCR3 in Hepa1-6 bearing LDRT treated mice (up panel) and quantification of % $TCF1^+$ PD-$1^+$ in $CD8^+$ T cells in the tumors of the indicated groups (down panel). Data of **(d)**, **(f)**, **(h)** and **(j)** shown as means ± SD derived from tumor mouse models (n=3 mice/group). Data of **(g)** and **(i)** shown as means ± SD of 3 independent biological experiments. *P* values of **(d)** and **(f-j)** were calculated using a two-sided unpaired Student's *t* test. Source data are provided as a Source Data file.

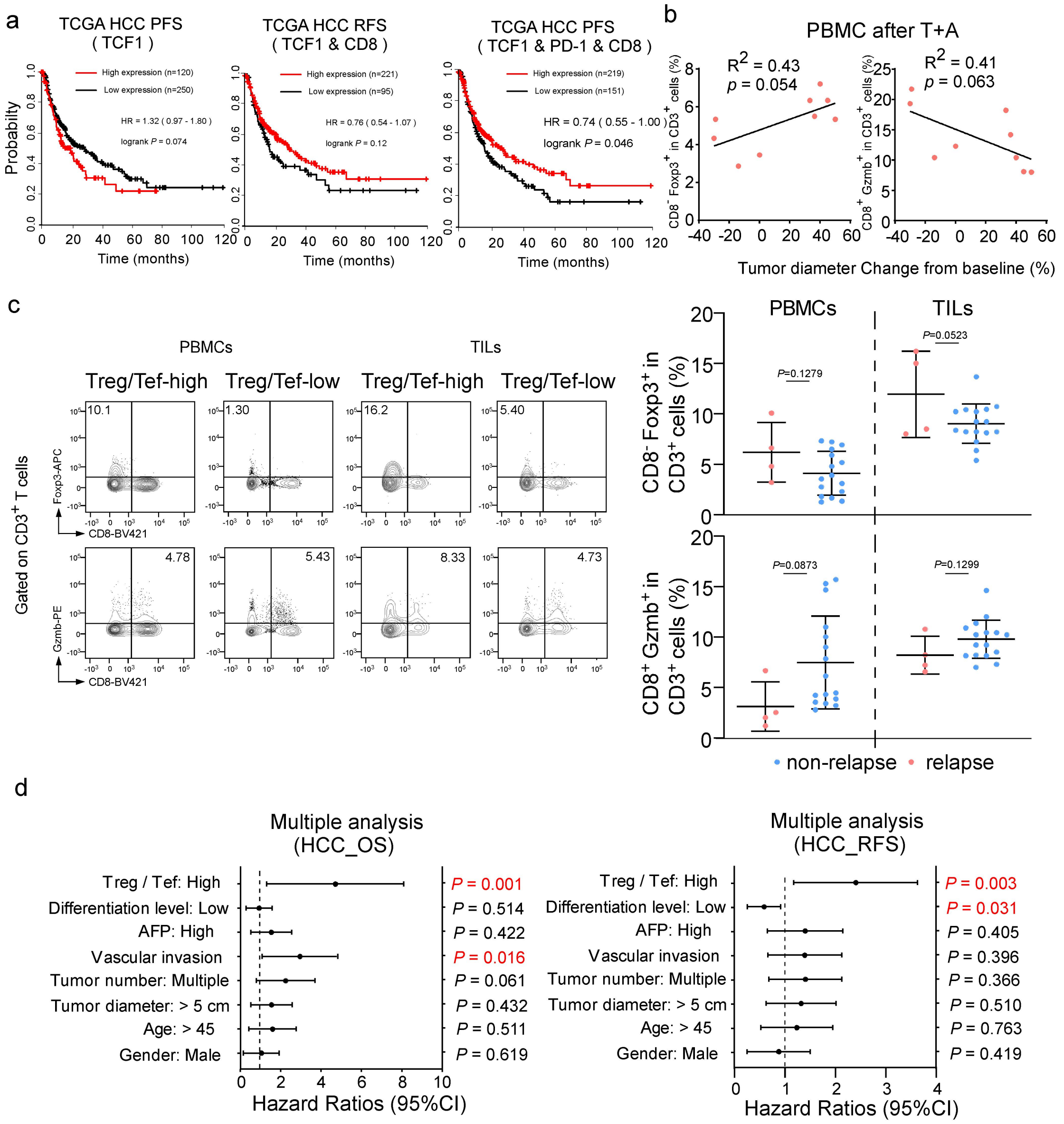


**Supplementary Fig. 12 Low infiltration of stem-like CD8+ Tpex and high Treg/Tef ratio correlates with poor prognosis in HCC.**

**(a)** The Kaplan–Meier Plotter (http://kmplot.com/analysis) program was used to analyze the infiltration of stem-like CD8+ Tpex in progress-free survival (PFS) of HCC. **(b)** Graphs depict correlation between the tumor diameter change from baseline (%) and Treg (%) or Tef (%). *P* value and $R^2$ value were calculated using linear regression analysis (n=9). **(c)** Representative flow cytometry plots (left panel) and quantification (right panel) of Treg/Tef of PBMC or TIICs in the indicated HCC groups. Relapse: n=4. non-relapse: n=16. *P* values were calculated using a two-sided unpaired Student's *t* test. Data of shown as means ± SD. **(d)** Multivariate Cox

regression analysis to evaluate the significance of the association between the Treg/Tef signature of 5-year OS and 5-year RFS in the presence of other clinical variables. Source data are provided as a Source Data file.

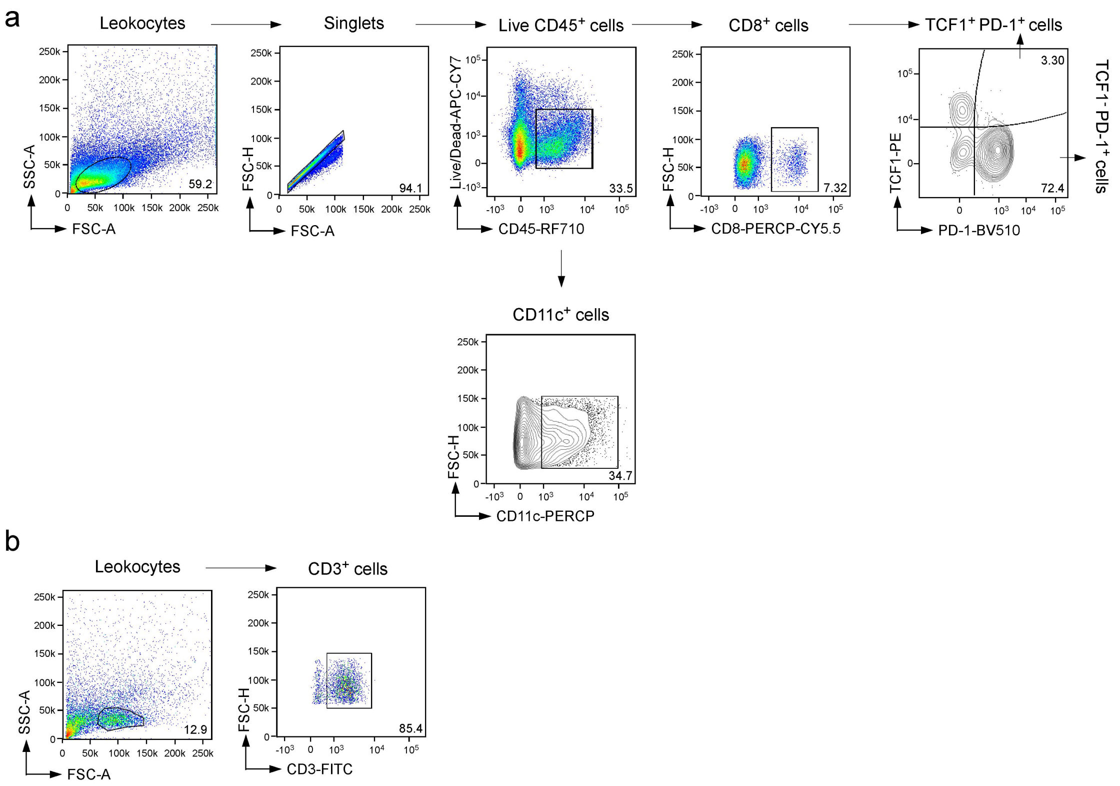


**Supplementary Fig. 13 Gating strategy.**

**(a)** Gating strategy used in Fig. 2, 4, 5, 7 and Supplementary Fig. 4, 5, 7, 9-11. **(b)** Gating strategy used in Fig. 7 and Supplementary Fig. 12.

**Supplementary Tables:**

**Supplementary Table 1. Clinical information of HCC patients with T+A treatment.**

| patient No. | $TCF1^+$ $PD1^+$ $CD8^+$ /$CD8^+$ cells (%) | Therapeutic effect of T+A |
|---|---|---|
| 1 | 4.16 | PR |
| 2 | 8.3 | PR |
| 3 | 10 | PR |
| 4 | 2.72 | SD |
| 5 | 2 | SD |
| 6 | 1.25 | PD |
| 7 | 1.76 | PD |
| 8 | 3 | SD |
| 9 | 2.67 | SD |

**Supplementary Table 2. Clinical information of HCC patients with T+A treatment.**

| patient No. | Therapeutic effect of T+A | change from baseline | Treg (%) in PBMC | Tef (%） in PBMC | Treg/Tef in PBMC |
|---|---|---|---|---|---|
| 1 | PR | -30% | 5.35 | 8.03 | 0.666251557 |
| 2 | PD | 50% | 6.35 | 8.12 | 0.782019704 |
| 3 | SD | -14% | 7.21 | 10.45 | 0.689952153 |
| 4 | PD | 36.40% | 5.51 | 14.21 | 0.387755102 |
| 5 | PD | 40% | 6.35 | 18.24 | 0.348135965 |
| 6 | PD | 33.30% | 3.46 | 12.29 | 0.281529699 |
| 7 | PD | 45% | 2.88 | 10.45 | 0.275598086 |
| 8 | PR | -29.10% | 5.35 | 21.75 | 0.245977011 |
| 9 | PD | 0% | 4.34 | 19.35 | 0.224289406 |

**Supplementary Table 3. Clinical information of primary HCC with surgical resection.**

| patient No. | recurrence status within 6 months | Treg (%) in PBMCs | Tef (%) in PBMCs | Treg/Tef in PBMCs | Treg (%) in TIICs | Tef (%) in TIICs | Treg/Tef in TIICs |
|---|---|---|---|---|---|---|---|
| 1 | 0 | 1.69 | 4.49 | 0.38 | 6.36 | 8.16 | 0.78 |
| 2 | 0 | 6.52 | 9.01 | 0.72 | 5.4 | 8.16 | 0.66 |
| 3 | 0 | 3.94 | 15.7 | 0.25 | 8.37 | 7.33 | 1.14 |
| 4 | 1 | 10.1 | 6.68 | 1.51 | 8.51 | 8.25 | 1.03 |
| 5 | 0 | 3.12 | 14.7 | 0.21 | 10.22 | 9.23 | 1.11 |
| 6 | 0 | 1.29 | 3.45 | 0.37 | 8.12 | 8.23 | 0.99 |
| 7 | 0 | 1.37 | 3.57 | 0.38 | 9.23 | 9.23 | 1.00 |
| 8 | 0 | 1.84 | 11 | 0.17 | 8.23 | 10.27 | 0.80 |
| 9 | 1 | 3.23 | 2.04 | 1.58 | 15 | 10.8 | 1.39 |
| 10 | 0 | 2.78 | 15.3 | 0.18 | 8.24 | 10.45 | 0.79 |
| 11 | 1 | 4.83 | 1.21 | 3.99 | 16.2 | 7.23 | 2.24 |
| 12 | 0 | 2.33 | 4.24 | 0.55 | 8.24 | 10.25 | 0.80 |
| 13 | 0 | 7.23 | 6.16 | 1.17 | 10.74 | 8.53 | 1.26 |
| 14 | 0 | 4.83 | 3.89 | 1.24 | 9.23 | 10.92 | 0.85 |
| 15 | 0 | 6.64 | 2.56 | 2.59 | 8.01 | 6.57 | 1.22 |
| 16 | 0 | 5.21 | 4.33 | 1.20 | 10.45 | 10.24 | 1.02 |
| 17 | 0 | 3.56 | 7.88 | 0.45 | 7.23 | 11.38 | 0.64 |
| 18 | 0 | 7.34 | 3.23 | 2.27 | 13.67 | 7.03 | 1.94 |
| 19 | 0 | 6.94 | 2.8 | 2.48 | 10.23 | 14.63 | 0.70 |
| 20 | 0 | 5.93 | 10.04 | 0.59 | 10.41 | 12.03 | 0.87 |

**Supplementary Table 4. Clinicopathological characteristics of 120 patients with primary HCC.**

| Parameters | Number of cases (%) |
|---|---|
| **Gender** | |
| Male | 103 (85.8) |
| Female | 17 (14.2) |
| **Age** | |
| ≤ 45 | 33 (27.5) |
| > 45 | 87 (72.5) |
| **Tumor diameter** | |
| ≤ 5 cm | 61 (50.8) |
| > 5 cm | 59 (49.2) |
| **Tumor number** | |
| Single | 81 (67.5) |
| Multiple | 39 (32.5) |
| **Vascular invasion** | |
| No | 70 (58.3) |
| Yes | 50 (41.7) |
| **AFP** | |
| < 400 ng/ml | 84 (70.0) |
| ≥ 400 ng/ml | 36 (30.0) |
| **Degree of differentiation** | |
| Intermediate and high | 41 (34.1) |
| Low | 79 (65.9) |
| **5-years vital status** | |
| Alive | 78 (65) |
| Dead | 42 (35) |
| **Relapse status within 5 years** | |
| No | 52 (43.3) |
| Yes | 68 (56.7) |
| **Treg/Tef ratio level** | |
| Low | 60 (50.0) |
| High | 60 (50.0) |

**Supplementary Table 5. Correlation between Treg/Tef and clinicopathological characteristics of patients with primary HCC.**

| Characteristics | Treg / Tef ratio level Low, no. of cases | High, no. of cases | *P* values |
|---|---|---|---|
| **Gender** | | | |
| Male | 51 | 52 | 0.793 |
| Female | 9 | 8 | |
| **Age** | | | |
| ≤ 45 | 19 | 14 | 0.307 |
| > 45 | 41 | 46 | |
| **Tumor diameter** | | | |
| ≤ 5 cm | 32 | 29 | 0.584 |
| > 5 cm | 28 | 31 | |
| **Tumor number** | | | |
| Single | 46 | 35 | **0.032** |
| Multiple | 14 | 25 | |
| **Vascular invasion** | | | |
| No | 39 | 31 | 0.139 |
| Yes | 21 | 29 | |
| **AFP** | | | |
| < 400 ng/ml | 45 | 39 | 0.232 |
| ≥ 400 ng/ml | 15 | 21 | |
| **Degree of differentiation** | | | |
| Intermediate and high | 18 | 23 | 0.336 |
| Low | 42 | 37 | |
| **5-years vital status** | | | |
| Alive | 51 | 27 | **<0.0001** |
| Dead | 9 | 33 | |
| **Relapse status within 5 years** | | | |
| No | 34 | 18 | **0.003** |
| Yes | 26 | 42 | |

*P* values were calculated using two-sided $\chi^2$ test.

**Supplementary Table 6. Univariate and multivariate analysis of factors associated with 5-year overall survival in patients with primary HCC.**

| Characteristics | Univariate analysis HR (95% CI) | Univariate analysis *P* values | Multivariate analysis HR (95% CI) | Multivariate analysis *P* values |
|---|---|---|---|---|
| **Gender** | | | | |
| Male *vs*. Female | 1.058 (0.446-2.511) | 0.899 | 0.788 (0.307-2.020) | 0.619 |
| **Age** | | | | |
| > 45 *vs*. ≤ 45 | 0.961 (0.492-1.877) | 0.907 | 1.306 (0.589-2.892) | 0.511 |
| **Tumor diameter** | | | | |
| > 5 cm *vs*. ≤ 5 cm | 1.541 (0.836-2.841) | 0.166 | 1.323 (0.658-2.658) | 0.432 |
| **Tumor number** | | | | |
| Multiple *vs*. Single | 2.178 (1.187-3.995) | **0.012** | 1.928 (0.970-3.831) | 0.061 |
| **Vascular invasion** | | | | |
| Yes *vs*. No | 3.23 (1.716-6.081) | **<0.0001** | 2.393 (1.180-4.856) | **0.016** |
| **AFP** | | | | |
| ≥ 400 ng/ml *vs*. < 400 ng/ml | 2.209 (1.202-4.059) | **0.011** | 1.314 (0.655-2.638) | 0.422 |
| **Degree of differentiation** | | | | |
| Intermediate and high *vs*. Low | 0.703 (0.306-.374) | 0.303 | 0.782 (0.373-1.639) | 0.514 |
| **Treg/Tef ratio level** | | | | |
| High *vs*. Low | 4.915 (2.347-10.291) | **<0.0001** | 3.888 (1.793-8.432) | **0.001** |

HR, hazard ratio; CI, confidence interval; Statistical analyses were performed using the Cox regression model (two-sided Likelihood ratio test).

**Supplementary Table 7. Univariate and multivariate analysis of factors associated with 5-year relapse-free survival in patients with primary HCC.**

| Characteristics | Univariate analysis | | Multivariate analysis | |
|---|---|---|---|---|
| | HR (95% CI) | *P* values | HR (95% CI) | *P* values |
| **Gender** | | | | |
| Male *vs.* Female | 1.045 (0.518-2.108) | 0.901 | 0.730 (0.341-1.564) | 0.419 |
| **Age** | | | | |
| > 45 *vs.* ≤ 45 | 0.890 (0.524-1.511) | 0.666 | 1.097 (0.601-2.004) | 0.763 |
| **Tumor diameter** | | | | |
| > 5 cm *vs.* ≤ 5 cm | 1.395 (0.866-2.248) | 0.171 | 1.200 (0.698-2.061) | 0.51 |
| **Tumor number** | | | | |
| Multiple *vs.* Single | 1.502 (0.917-2.460) | 0.106 | 1.280 (0.750-2.184) | 0.366 |
| **Vascular invasion** | | | | |
| Yes *vs.* No | 1.685 (1.046-2.715) | **0.032** | 1.266 (0.734-2.182) | 0.396 |
| **AFP** | | | | |
| ≥ 400 ng/ml *vs.* < 400 ng/ml | 1.615 (0.976-2.613) | 0.062 | 1.266 (0.727-2.202) | 0.405 |
| **Degree of differentiation** | | | | |
| Intermediate and high *vs.* Low | 0.556 (0.327-0.945) | **0.03** | 0.524 (0.291-0.943) | **0.031** |
| **Treg/Tef ratio level** | | | | |
| High *vs.* Low | 2.260 (1.384-3.691) | **0.001** | 2.195 (1.295-3.720) | **0.003** |

HR, hazard ratio; CI, confidence interval; Statistical analyses were performed using the Cox regression model (two-sided Likelihood ratio test).

**Supplementary Table 8. The antibodies used in flow cytometry.**

| Antibody / Dye | Brand | Cat# | Dilutions | clone numbers |
|---|---|---|---|---|
| Ghost Dye Red780 | TONBO Bioscience | 13-0865-T100 | 1 ul/1 ml | NA |
| anti-mouse CD45-redFluor 710 | TONBO Bioscience | 80-0451-U100 | 0.25 µg per $10^6$ cells in 100 µl | 30-F11 |
| anti-mouse CD4-APC | Biolegend | 100411 | 0.25 µg per $10^6$ cells in 100 µl | GK1.5 |
| anti-mouse CD8-PerCP-Cy5.5 | TONBO Bioscience | 65-0081-U025 | 0.25 µg per $10^6$ cells in 100 µl | 53-6.7 |
| anti-mouse TNF-α-BV421 | Biolegend | 506328 | 0.25 µg per $10^6$ cells in 100 µl | MP6-XT22 |
| anti-mouse PD-1-BV510 | Biolegend | 135241 | 0.25 µg per $10^6$ cells in 100 µl | 29F.1A12 |
| anti-mouse KI-67-FITC | eBioscience | 11-5698-80 | 0.25 µg per $10^6$ cells in 100 µl | SolA15 |
| anti-mouse CD11b-PE | Biolegend | 101207 | 0.25 µg per $10^6$ cells in 100 µl | M1/70 |
| anti-mouse CD86-APC-CY7 | Biolegend | 105030 | 0.25 µg per $10^6$ cells in 100 µl | GL-1 |
| anti-mouse CD11c-PERCP | Biolegend | 117325 | 0.25 µg per $10^6$ cells in 100 µl | N418 |
| anti-mouse MHC-I-APC | Biolegend | 114713 | 0.25 µg per $10^6$ cells in 100 µl | 34-1-2S |
| anti-mouse IFN-γ-PE-Cy7 | TONBO Bioscience | 60-7311-U100 | 0.125 µg per $10^6$ cells in 100 µl | XMG1.2 |
| anti-mouse SLAMF6-PE | Biolegend | 134606 | 1.0 µg per $10^6$ cells in 100 µl | 330-AJ |
| anti-mouse PRF1-APC | Invitrogen | 17-9392-80 | 1.0 µg per $10^6$ cells in 100 µl | 17-9392-80 |

| | | | | |
|---|---|---|---|---|
| anti-mouse TCF1-PE | CST | 14456S | 1:50 | C63D9 |
| anti-mouse F4/80-APC | Biolegend | 157306 | 0.5 µg per $10^6$ cells in 100 µl | QA17A29 |
| anti-mouse CD206-PERCP-CY5.5 | Biolegend | 141716 | 0.5 µg per $10^6$ cells in 100 µl | C068C2 |
| anti-mouse CXCR3-BV421 | Biolegend | 126521 | 0.5 µg per $10^6$ cells in 100 µl | CXCR3-173 |
| anti-mouse GZMB-FITC | Biolegend | 372206 | 5 µl per $10^6$ cells in 100 µl | QA16A02 |
| anti-human CD3-FITC | Biolegend | 317306 | 5 µl per $10^6$ cells in 100 µl | OKT3 |
| anti-human CD8-BV421 | Biolegend | 301035 | 5 µl per $10^6$ cells in 100 µl | RPA-T8 |
| anti-human GZMB-PE | eBioscience | 12-8899-41 | 5 µl per $10^6$ cells in 100 µl | GB11 |
| anti-human FOXP3-APC | eBioscience | 17-4776-42 | 5 µl per $10^6$ cells in 100 µl | 1704776-42 |
| anti-human PD-1-PERCP | Biolegend | 329937 | 5 µl per $10^6$ cells in 100 µl | EH12.2H7 |
| anti-human TCF1-PE | Biolegend | 655208 | 5 µl per $10^6$ cells in 100 µl | 7F11A10 |
