# Supplementary material for: Low-dose radiotherapy combined with dual PD-L1 and VEGFA blockade elicits antitumor response in hepatocellular carcinoma mediated by activated intratumoral CD8+ exhausted-like T cells
Source: Nat Commun. 2023 Nov 24;14:7709. doi: 10.1038/s41467-023-43462-1 (PMC10673920; doi:10.1038/s41467-023-43462-1)
Supplement: Supplementary file 3 — Reporting Summary [file 41467_2023_43462_MOESM3_ESM.pdf]

nature portfolio

Corresponding author(s): Hua Li, Shuqun Cheng, Linsen Ye, Yang Yang

Last updated by author(s): Nov 2, 2023

Reporting Summary

Nature Portfolio wishes to improve the reproducibility of the work that we publish. This form provides structure for consistency and transparency in reporting. For further information on Nature Portfolio policies, see our Editorial Policies and the Editorial Policy Checklist.

Statistics

For all statistical analyses, confirm that the following items are present in the figure legend, table legend, main text, or Methods section.

n/a	Confirmed	
☐	☒	The exact sample size (n) for each experimental group/condition, given as a discrete number and unit of measurement
☐	☒	A statement on whether measurements were taken from distinct samples or whether the same sample was measured repeatedly
☐	☒	The statistical test(s) used AND whether they are one- or two-sided<br>*Only common tests should be described solely by name; describe more complex techniques in the Methods section.*
☐	☒	A description of all covariates tested
☐	☒	A description of any assumptions or corrections, such as tests of normality and adjustment for multiple comparisons
☐	☒	A full description of the statistical parameters including central tendency (e.g. means) or other basic estimates (e.g. regression coefficient) AND variation (e.g. standard deviation) or associated estimates of uncertainty (e.g. confidence intervals)
☐	☒	For null hypothesis testing, the test statistic (e.g. F, t, r) with confidence intervals, effect sizes, degrees of freedom and P value noted<br>*Give P values as exact values whenever suitable.*
☒	☐	For Bayesian analysis, information on the choice of priors and Markov chain Monte Carlo settings
☒	☐	For hierarchical and complex designs, identification of the appropriate level for tests and full reporting of outcomes
☒	☐	Estimates of effect sizes (e.g. Cohen's d, Pearson's r), indicating how they were calculated

Our web collection on statistics for biologists contains articles on many of the points above.

Software and code

Policy information about availability of computer code

Data collection	Flow cytometry data was acquired on a BD LSRFortessa cytometer. The multiplex immunohistochemistry images were acquired on the tissue FAXS SL spectra (TissueGnostics, Austria).
Data analysis	Serum data were analyzed by an automatic analyzer (Hitachi 3100, Japan). Single-cell data were mainly processed by the R package Seurat (v.4.3.0). Flow cytometry data was analyzed using FlowJo software (v10). The RNA sequencing was performed on Dr. Tom Multi-omics Data system (https://biosys.bgi.com). The multiplex immunohistochemistry images were analyzed using tissueFAXS SL viewer. The immunofluorescence staining was evaluated by ImageJ. GraphPad Prism V.7 and IBM SPSS Statistics V.20 were used to analyze the data.<br><br>R scripts for Seurat processing are available on GitHub [https://github.com/satijalab/seurat]. ProjecTILs is available on GitHub [https://github.com/carmonalab/ProjecTILs]. Monocle2 is available at [https://cole-trapnell-lab.github.io/monocle-release].

For manuscripts utilizing custom algorithms or software that are central to the research but not yet described in published literature, software must be made available to editors and reviewers. We strongly encourage code deposition in a community repository (e.g. GitHub). See the Nature Portfolio guidelines for submitting code & software for further information.

Data

Policy information about availability of data

All manuscripts must include a data availability statement. This statement should provide the following information, where applicable:

- Accession codes, unique identifiers, or web links for publicly available datasets
- A description of any restrictions on data availability
- For clinical datasets or third party data, please ensure that the statement adheres to our policy

The raw data from four groups of scRNA-seq for mouse tumor infiltrating immune cells and RNA-seq for mouse tumor tissues in Hepa1-6 tumors generated in this study have been deposited in the Genome Sequence Archive (GSA) database under accession code CRA011374 [https://bigd.big.ac.cn/gsa/browse/CRA011374]. The raw data from four groups of scRNA-seq for mouse CD45+ tumor infiltrating immune cells s in DEN+CCI4 tumors generated in this study have been deposited in the Genome Sequence Archive (GSA) database under the accession code CRA012644 [https://bigd.big.ac.cn/gsa/browse/CRA012644]. The Kaplan-Meier Plotter [http://kmplot.com/analysis] publicly available data used in this study are available in The Cancer Genome Atlas (TCGA) database (project TCGA-LIHC) under dbGaP Study Accession phs000178 [https://cancergenome.nih.gov/]. The remaining data are available within the Article, Supplementary Information or Source Data file. Source data are provided with this paper.

Research involving human participants, their data, or biological material

Policy information about studies with human participants or human data. See also policy information about sex, gender (identity/presentation), and sexual orientation and race, ethnicity and racism.

Reporting on sex and gender	The sex and gender of patients were not specifically considered in this study. PDTFs were established from 6 male HCC patients. HCC biopsy tissues from T+A treated patients were collected from 7 male and 2 female patients. HCC patients recruited for prognostic analysis were consist of 103 male and 17 female individuals.
Reporting on race, ethnicity, or other socially relevant groupings	No information/data about race, ethnicity or other socially relevant groupings were collected.
Population characteristics	HCC tumor tissues used for PDTFs were acquired from 6 HCC patients, aged between 34 and 61 years, who underwent open hepatectomy at the Third Affiliated Hospital of Sun Yat-sen University. HCC tumor biopsy specimens were collected from 9 patients with HCC, aged between 43 and 83 years, at the Eastern Hepatobiliary Surgery Hospital, Naval Medical University, for multiplex immunohistochemistry (mIHC) analysis. Fresh blood samples were collected from 9 HCC patients, aged between 45 and 64 years, after T+A treatment in the Third Affiliated Hospital of Sun Yat-sen University. Fresh clinical tumor samples and blood samples were acquired from 20 HCC patients, aged between 38 and 85 years, who underwent open hepatectomy at the Third Affiliated Hospital of Sun Yat-sen University. This study used 120 paraffin-embedded HCC patient specimens that had been clinically and histopathologically diagnosed at the the Third Affiliated Hospital of Sun Yat-sen University from January 2011 to December 2017 and details of the covariate-related population characteristics of human research participants (such as age, gender, etc,) were provided in the Supplementary Table 4.
Recruitment	There are no potential self-selection bias or other biases present in our study.
Ethics oversight	Clinical specimens were used for research purposes with prior patient consent and ethical approval from the institutional research ethics committees of Third Affiliated Hospital of Sun Yat-sen University (ethics approval number: II2023-068-01) and the Ethics Committee of Eastern Hepatobiliary Surgery Hospital, Naval Medical University (ethics approval number: EHBHKY2021-K-017).

Note that full information on the approval of the study protocol must also be provided in the manuscript.

Field-specific reporting

Please select the one below that is the best fit for your research. If you are not sure, read the appropriate sections before making your selection.

☒ Life sciences ☐ Behavioural & social sciences ☐ Ecological, evolutionary & environmental sciences

For a reference copy of the document with all sections, see nature.com/documents/nr-reporting-summary-flat.pdf

Life sciences study design

All studies must disclose on these points even when the disclosure is negative.

Sample size	The sample size for the in vivo studies to achieve statistical significance was not calculated before the studies as the efficiency of T+A and DPVB treatments in the different models was unknown prior. Sample sizes in vivo and vitro were determined by three or more samples for comparisons between one or multiple groups, followed by the statistical test.
Data exclusions	No pre-established data exclusion method was used.
Replication	The experimental findings can be reliably reproduced. Some key data generated by one co-author were repeated by other co-authors. The figure legends specify how often the experiments were replicated or performed independently.

Randomization	All samples were number coded until the readout was finalized. The numbers were assigned prior to the experiment and determined the group/ treatment/ condition. Animals were number coded and randomly assigned to a group prior to the treatment. For IHC,mIHC and IF assays, 5 fields of view were randomly selected for each slice.
Blinding	Mice group allocation were performed by blinded investigators. The experimental results of molecular and cellular biology are obtained by objective quantitative methods(PMCID: PMC8438024), so we were not blinded to sample allocation.

Reporting for specific materials, systems and methods

We require information from authors about some types of materials, experimental systems and methods used in many studies. Here, indicate whether each material, system or method listed is relevant to your study. If you are not sure if a list item applies to your research, read the appropriate section before selecting a response.

Materials & experimental systems

n/a	Involved in the study
☐	☒ Antibodies
☐	☒ Eukaryotic cell lines
☒	☐ Palaeontology and archaeology
☐	☒ Animals and other organisms
☒	☐ Clinical data
☒	☐ Dual use research of concern
☒	☐ Plants

Methods

n/a	Involved in the study
☒	☐ ChIP-seq
☐	☒ Flow cytometry
☒	☐ MRI-based neuroimaging

Antibodies

Antibodies used

Antibodies used for in vivo studies are listed as follows:
Rat IgG2b isotype control-InVivo (Selleck Cat# A2116), anti-mouse CD8α-InVivo (Selleck Cat# A2102), anti-mouse CD4-InVivo (Selleck Cat# A2101), InVivoMAb polyclonal Armenian hamster IgG (BioXCell, Cat# BE0091), InVivoMAb anti-mouse CXCR3 (CD183) (BioXCell, Cat# BE0249).
Antibodies used for flow cytometry are listed as follows:
Ghost Dye Red780 TONBO Bioscience 13-0865-T100
anti-mouse CD45-redFluor 710 TONBO Bioscience 80-0451-U100 30-F11
anti-mouse CD4-APC Biolegend 100411 GK1.5
anti-mouse CD8-PerCP-Cy5.5 TONBO Bioscience 65-0081-U025 53-6.7
anti-mouse TNF--BV421 Biolegend 506328 MP6-XT22
anti-mouse PD-1-BV510 Biolegend 135241 29F.1A12
anti-mouse KI-67-FITC eBioscience 11-5698-80 SolA15
anti-mouse CD11b-PE Biolegend 101207 M1/70
anti-mouse CD86-APC-CY7 Biolegend 105030 GL-1
anti-mouse CD11c-PERCP Biolegend 117325 N418
anti-mouse MHC-I-APC Biolegend 114713 34-1-2S
anti-mouse IFN--PE-Cy7 TONBO Bioscience 60-7311-U100 XMG1.2
anti-mouse SLAMF6-PE Biolegend 134606 330-AJ
anti-mouse PRF1-APC Invitrogen 17-9392-80 17-9392-80
anti-mouse TCF1-PE CST 14456S C63D9
anti-mouse F4/80-APC Biolegend 157306 QA17A29
anti-mouse CD206-PERCP-CY5.5 Biolegend 141716 C068C2
anti-mouse CXCR3-BV421 Biolegend 126521 CXCR3-173
anti-mouse GZMB-FITC Biolegend 372206 QA16A02
anti-human CD3-FITC Biolegend 317306 OKT3
anti-human CD8-BV421 Biolegend 301035 RPA-T8
anti-human GZMB-PE eBioscience 12-8899-41 GB11
anti-human FOXP3-APC eBioscience 17-4776-42 1704776-42
anti-human PD-1-PERCP Biolegend 329937 EH12.2H7
anti-human TCF1-PE Biolegend 655208 7F11A10
The antibodies used in IHC：
CD4 (abcam, EPR19514, Cat# ab183685, 1:1000), CD8 (abcam, EPR21769, Cat# 217344, 1:2000).secondary antibody (Dako, Cat# K5007).
The antibodies used in mIHC：
TCF1 (CST, C63D9, Cat# 2203S, 1:200), PD-1 (CST, D4W2J, Cat# 86163S, 1:200) and CD8 (abcam, C8/468 + C8/144B, Cat# ab199016, 1:200). horseradish peroxidase-conjugated secondary antibody
The antibodies used in IF:
CD8 (proteintech, 1G2B10, Cat# 66868-1-1g, 1:400) and GZMB (abcam, EPR22645-206, Cat# ab255598, 1:3000), CD4 (abcam,

	EPR6855, Cat# ab133616,1:500) and FOXP3 (abcam, mAbcam 22510, Cat# ab22510, 1:100). secondary antibodies: Cy3 Goat-Anti-Rabbit IgG (H+L) Antibody (APE x BIO, Cat# K1209) or FITC Goat-Anti-Mouse IgG (H+L) Antibody (APE x BIO, Cat# K1201)
Validation	The antibodies utilized in this study were commercially sourced and have undergone validation by their respective manufacturers, with the validation data accessible on the corresponding websites. Prior to usage, all flow panels underwent thorough validation.

Eukaryotic cell lines

Policy information about cell lines and Sex and Gender in Research

Cell line source(s)	The Hepa1-6 cell line were obtained from ATCC.
Authentication	Cell lines were not authenticated.
Mycoplasma contamination	The cell lines were regularly tested negative for mycoplasma.
Commonly misidentified lines (See ICLAC register)	No misidentified lines were used.

Animals and other research organisms

Policy information about studies involving animals; ARRIVE guidelines recommended for reporting animal research, and Sex and Gender in Research

Laboratory animals	Two/six/eight-week-old male C57BL/6J mice were purchased from the Model Animal Research Center of Nanjing University (China). All mice were raised under specific pathogen-free (SPF) conditions with restrict 12 hours day/night cycle at temperature 18-22°C and humidity 50-60%.
Wild animals	No wild animals were used
Reporting on sex	The study did not involve.
Field-collected samples	No field collected samples were used.
Ethics oversight	The animal research in this study were approved by the Institutional Animal Care and Use Committee of the South China Agricultural University.

Note that full information on the approval of the study protocol must also be provided in the manuscript.

Flow Cytometry

Plots

Confirm that:

- [x] The axis labels state the marker and fluorochrome used (e.g. CD4-FITC).
- [x] The axis scales are clearly visible. Include numbers along axes only for bottom left plot of group (a 'group' is an analysis of identical markers).
- [x] All plots are contour plots with outliers or pseudocolor plots.
- [x] A numerical value for number of cells or percentage (with statistics) is provided.

Methodology

Sample preparation	Tumor samples were dissociated in 1640 medium containing 10% fetal bovine serum (FBS) and 1% 100X penicillin-streptomycin solution (P/S) supplemented with 0.5 mg/ml of collagenase-IV (Sigma-Aldrich, Cat# C5138) and 0.1 mg/ml of DNase-I (Sigma-Aldrich, Cat# DN25) at 37°C for 1 hour. Uniform single-cell suspensions were obtained after smashing digested tissues with a syringe plunger on a 40 mm filter. Then, differential speed centrifugation to isolate tumor infiltrated lymphocytes (TILs) from the single-cell suspensions by mouse lymphocyte isolation medium (Dayou, Cat# 7211011) or percoll (Pharmacia (GE), Cat# 17089109-1). The protocol used for preparation of single cells of dLNs were obtained of smashing tissues with a syringe plunger on a 40 m m filter. Human peripheral blood lymphocyte isolation medium (TBD, Cat# LTS1077) centrifugation was used to isolate peripheral blood mononuclear (PBMC) from blood samples.
Instrument	Fluorescence data were acquired on a BD LSRFortessa cytometer.
Software	Fluorescence data were analyzed using FlowJo V.X.
Cell population abundance	For flow cytometry analysis, 10000 cells were measured.

Gating strategy

Samples are gated in FSC/SSC for correct cell size and live cells. The detail gating strategy was present under the plots.

☒ Tick this box to confirm that a figure exemplifying the gating strategy is provided in the Supplementary Information.
